# Successful Outcome of Patellectomy Plus Chemotherapy for Primary Bone Lymphoma of the Patella: A Case Report and Literature Review

**DOI:** 10.3389/fonc.2021.786495

**Published:** 2021-12-13

**Authors:** Xin Cao, Hui-Jin Chen

**Affiliations:** ^1^ Department of Traumatic Orthopedics, Shengli Oilfield Central Hospital, Dongying, China; ^2^ Department of Clinical Laboratory, Shengli Oilfield Central Hospital, Dongying, China

**Keywords:** primary bone lymphoma, lymphoma, patella, patellectomy, chemotherapy

## Abstract

Primary bone lymphoma (PBL) is a rare but distinct clinicopathological disease, usually occurring in the pelvis, spine, and ribs. To date, only a few cases have been reported as beginning in the patella. Due to the lack of clinical evidence, the optimal treatment strategy has not been established. Here, we report a case that presented unexplained right knee pain. The case was diagnosed with the non-germinal center, diffuse large B cell lymphoma in the patella by imaging examinations and bone biopsy. Then, the patient received a patellectomy and eight cycles of R-CHOP chemotherapy. After treatment, the patient achieved a favorable prognosis and satisfactory functional recovery.

## Introduction

Primary bone lymphomas (PBLs) are the rare and peculiar extranodal presentation of non-Hodgkin’s lymphomas (NHLs), usually occurring in the pelvis, spine, and ribs (1). PBLs beginning in the patella are extremely rare. To date, only six cases with PBL in the patella have been reported (2–7). However, treatment methods in these reports were diversified and resulted in various prognoses; more critically, there was no follow-up evaluation for the knee joint function. Due to the unspecific imaging findings and the low morbidity, there is no unified diagnosis and treatment standard for this disease. Here, we reported a case diagnosed with diffuse large B cell lymphoma in the patella and the successful treatment outcome. Our patient was reported to provide evidence for the standard of care.

## Case Report

A 50-years-old female presented with right-knee pain and a limited range of motion when upstairs. She denied any significant medical and malignancy history. The patient complained of mild, persisted, localized pain in the right knee with unexplained causes. The persistent pain was considered to be due to patellofemoral osteoarthritis; thus, she had received an analgesic. Physical examination confirmed the limited range of motion.

**Figure 1:**
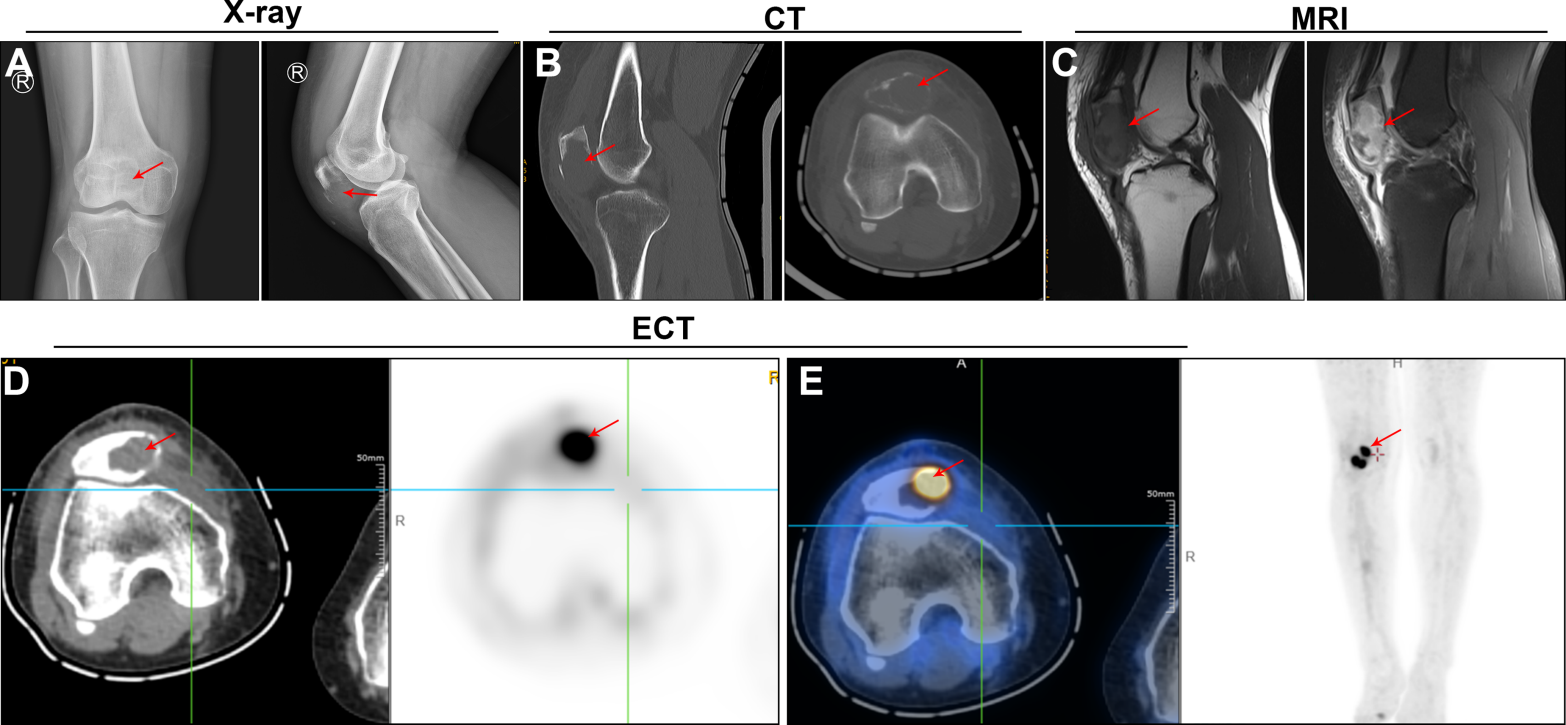
Imaging views of the knee. **(A)** Anterior and lateral X-ray views showed the osteolytic changes (red arrows) in the patella. **(B)** Sagittal and transverse computed tomography (CT) images showed the decreased bone density (red arrows) in the patella, and tumor involvement inside the whole patella. **(C)** Sagittal magnetic resonance imaging (MRI) revealed a low signal in the T1-weighted image and a high signal in fat-suppressed T2-weighted image (red arrows). **(D**, **E)** Positron emission tomography-computed tomography (PET/CT) showed extremely intense focal uptake in the right knee joint and no focal uptake in other sites.

X-ray examination revealed the decreased bone density, cortical bone discontinuity, slight swelling, and unchanged knee joint space of the right patella, which confirmed a pathologic fracture of the right patella (**Figure 1A**). The radiological image exhibited osteolytic lesions in the patella, raising suspicion of a patellar tumor (**Figure 1A**). Further workup was ordered, including computed tomography (CT) scan, magnetic resonance imaging (MRI), and positron emission tomography-CT (PET/CT). CT scan showed the tumor involvement inside the whole patella and the lesion with defining borders (**Figure 1B**). MRI revealed that the lesion had a decreased signal in the T1-weighted image (T1WI), as well as a high signal in fat-suppressed T2-weighted image (T2WI-FS) with surrounding soft-tissue swelling and fluid effusion in the articular cavity (**Figure 1C**). In the SPECT/CT fusion image, an extensive osteolytic area on the left side of the patella was observed (**Figure 1D**). Further, a whole-body PET/CT scan showed extremely intense focal uptake in the right knee joint and no focal uptake in other sites (**Figure 1E**).

**Figure 2:**
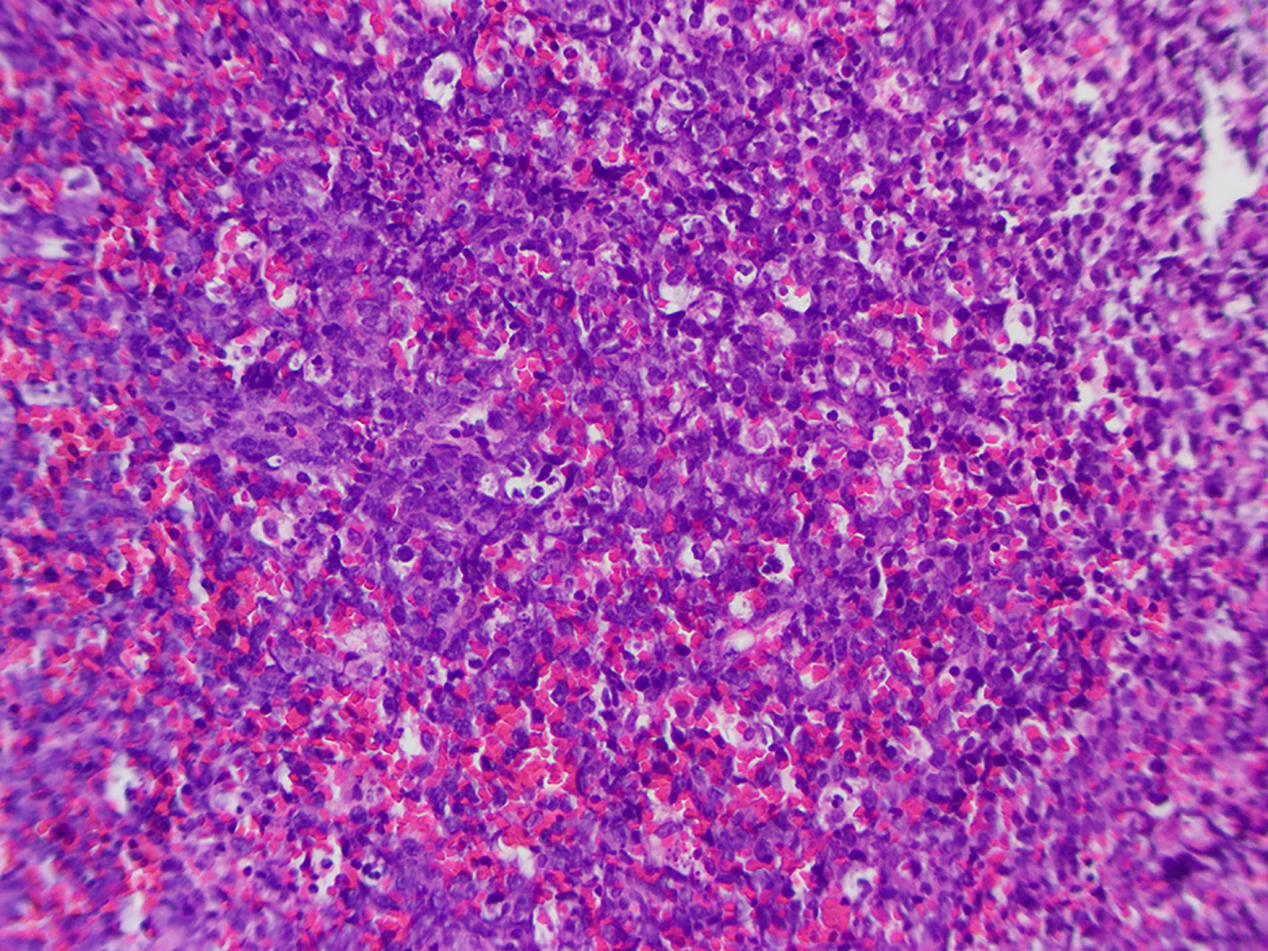
Histologic examination of the right patellar mass reveals the diffuse infiltration by atypical lymphocytes.

On the fifth day of hospitalization, an open biopsy of the patellar lesion was performed. A semi-transparent, jelly-like mass was observed during the biopsy. Subsequently, the pathologic examination of the patella demonstrated the diffuse infiltration by atypical lymphocytes, which was highly pleomorphic (**Figure 2**). Immunohistochemistry (IHC) staining showed that tumor cells were CD20, CD79a, LCA, c-Myc (30%), Bcl-2, H3K27me3, and MUM-1 positive, but were negative for CK broad-spectrum, Vimentin, EMA, HMB45, CD3, CD5, CD21, CD30, CD38, CD56, CD68, CD10, CD117, S-100, CD117, cyclinD1, Bcl-6, CR, CXCL-13, desmin, STAT-6, ERG, langerin, lysozyme, myoglobin, myo-D1, TLE-1, lappa, and lambda. Ki-67 proliferation index was high (75–80%). Based on these pathological findings, a diagnosis of non-germinal center, diffuse large B cell lymphoma (DLBCL) was made. According to the imaging results, the tumor was considered stage IE of the Ann Arbor classification. The International Prognostic Index (IPI) score was 0, suggesting that this is a low-risk tumor.

**Figure 3:**
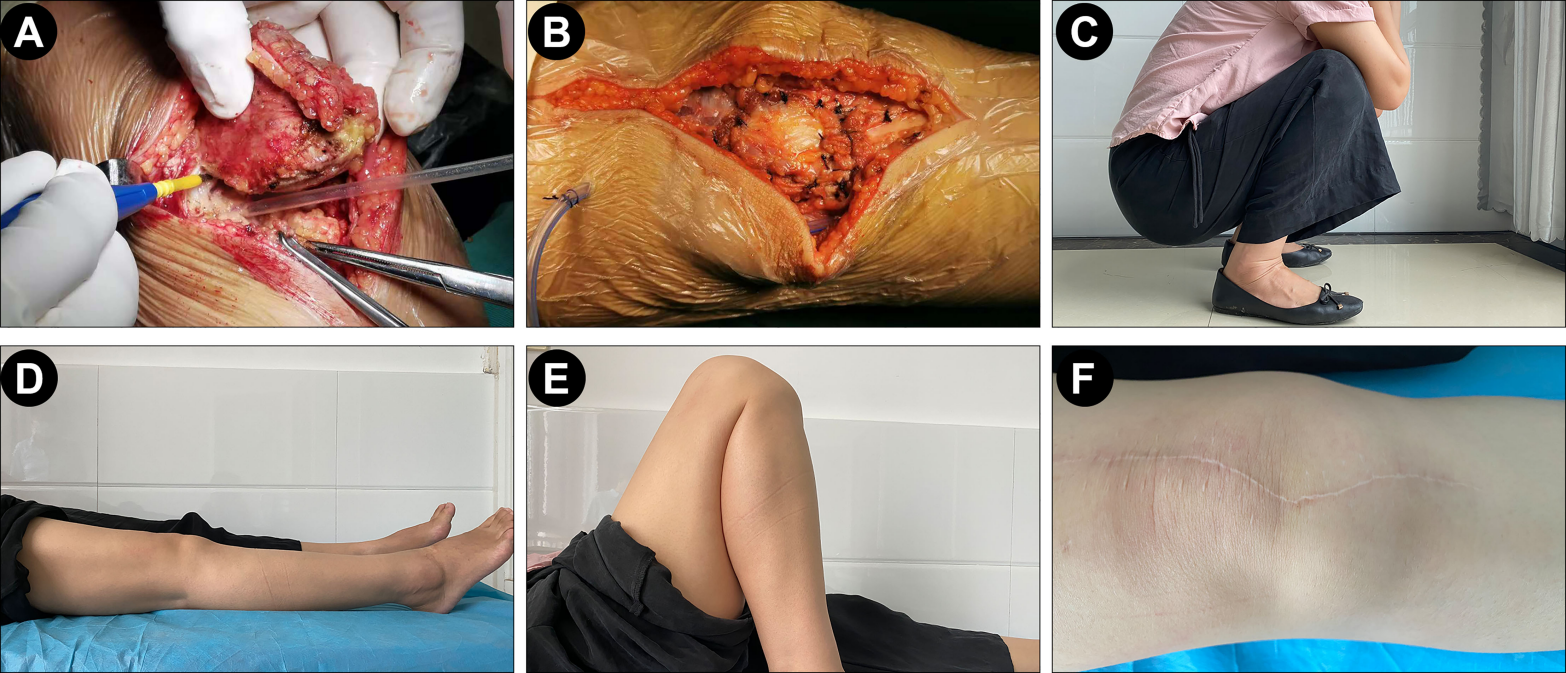
Surgical operation and post-operation follow-up. **(A, B)** The expansion of quadriceps femoris muscle and the residual support structure surrounding the patella was directly sutured after the patella was completely resected. **(C–F)** Postoperative functional examination demonstrated a satisfactory functional recovery of the patient.

Considering that the tumor is primary lymphoma arising in the patella, the patient underwent a patellectomy. The surgical procedure demonstrated the involvement of the patella interior and no invasion of the surrounding tissue (**Figures 3A, B**). Postoperatively, the affected limb was fixed with an extension brace for 6 weeks. After removing the brace, the patient received functional and weight-loaded walking training. Meanwhile, the patient received eight cycles of rituximab plus cyclophosphamide, doxorubicin, vincristine, and prednisone (CHOP) regimen (R-CHOP) chemotherapy. After six cycles of chemotherapy, PET/CT scan showed that only the soft-tissue in the surgical site was thickening with hypermetabolic [18F]-fluorodeoxyglucose (FDG) uptake, but no abnormal changes in other sites. MRI examination revealed a slight soft-tissue swelling in the right patella and a small fluid effusion in the articular cavity. At the 1-year follow-up clinic visit, the patient had completed all therapy and achieved a satisfactory functional recovery (**Figures 3C–F**). No functional limitation (defined as a flexion/extension muscle strength <4 levels) was observed when the knee joint flexed and extended. Postoperative functional examination demonstrated that the knee joint flexion/extension muscle strength was at five levels, and the American Knee Society (AKS) score was 95 points. The range of the joint motion of the right knee returned to 0–135°. The chronic pain resolved. However, she occasionally presented only mild pain and slight swelling in the anterior region of the knee. The patient claimed that she could walk and stand for a long period. Radiological re-examination showed no recurrence or metastases.

## Discussion

PBLs are rare extranodal malignant lymphoma derived from the marrow cavity (8, 9). However, PBL occurring from the patella is very rare. Until now, only 6 cases with PBL in the patella were reported previously (**Table 1**) (2–7). Given the limited cases and incomplete case details, there is no comprehensive overview of the diagnosis, treatment, prognosis, and patellar functionality follow-up of PBL involving the patella.

**Table 1:** Details of the reported primary bone lymphoma of patella.

Author, year	Age	Sex	Clinical symptom	Radiological findings			Pathologic findings	Diagnosis	Treatment	Prognosis
				X-ray	MRI	PET/CT				
Chandra (2)	52	Female	Knee pain, swelling	Osteolysis; pathological fracture	Not available.	No other sites of involvement.	CD45^+^; CD20^+^; intermediate Ki-67 PI.	LBCL	CHOP chemotherapy; radiation	Complete remission 13 months later.
Hughes (3)	76	Male	Knee pain, stiff, swollen	Osteolysis; pathological fracture	Not available.	Increased uptake within the patella; no other sites of involvement.	Not available.	Non-Hodgkin’s lymphoma	Patellectomy; chemotherapy	Local and nodal recurrence within the femoral region on the involved side; poor prognosis.
Agarwal (4)	72	Male	Knee pain, subcutaneous nodules	Osteolysis	Not available.	Not available.	CD3^+^; CD45^+^; CD19^−^; CD30^−^; diffuse infiltration.	Primary T-cell lymphoma	CHOP chemotherapy	Subcutaneous nodules and knee swelling subsided.
Wills (5)	71	Male	Knee pain, swelling; ROM 25–120°	Erosive changes; soft tissue mass; pathological fracture	Erosive changes with abnormal signal changes.	No other sites of involvement.	CD20^+^; CD79a^+^; Bcl-6^+^; CD10^+^; Ki-67 PI 60%.	DLBCL, germinal center subtype	Iliac crest grafting; chemotherapy	Chronic pain had resolved; ROM symmetric to contralateral knee; No recurrence or metastases.
Yamamoto (7)	56	Female	Knee pain, swelling; ROM 0–50°	Osteolysis; pathological fracture	T1WI, moderate intensity; T2WI, high intensity.	Abnormal uptake of FDG in the patella.	CD20^+^; CD79a^+^; MUM-1^+^; Bcl-6^−^; CD3^−^; CD10^−^.	DLBCL, non-germinal center subtype	R-CHOP chemotherapy; radiation	Recurrence and metastases; died of disease 3.5 years after the initial onset.
Jadidi (6)	58	Female	Knee pain, limited ROM	Osteolysis	T1WI, decreased signal; STIR, increased signal and avid contrast enhancement	Lytic lesion with ill-defining borders; hypermetabolic FDG uptake within the left patella and popliteal fossa	CD45^+^; CD2^+^; CD3^+^; CD4^+^; CD5^+^; CD8^+^; CD25^+^; FOXP3^+^; CD7 (partial loss); Ki-67 PI 60–70%; HTLV-I^+^	Primary T-cell lymphoma	Planed chemoradiotherapy	Not available.

LBCL, large B-cell lymphoma; DLBCL, diffuse large B-cell lymphoma; HTLV-I, Human T-cell Lymphotropic Virus-I; FDG, [18F]-fluorodeoxyglucose; PI, proliferative index; ROM, range of motion; CHOP, cyclophosphamide, doxorubicine, vincristine, and prednisone; R-CHOP, rituximab plus CHOP.

Clinically, based on the previous evidence, the most common symptoms of patellar tumors manifest as swelling and pain in the anterior region of the knee, as seen in our case. Meanwhile, the pain is usually non-specific and persists for several months or years. This disease is frequently misdiagnosed with knee osteoarthritis because it has similar clinical symptoms to the patellar tumor. Typical X-ray findings of PBLs include osteolytic lesions (70%), osteogenic lesions (2–5%), and mixed lesions (20–30%) (10, 11). Consistently, our patient presented the typical osteolytic destruction in the X-ray image. Besides, the MRI presentations of our case are also consistent with the published cases (5–7), that is, low intensity in T1WI and high intensity in T2WI-FS. In the PET/CT images, we observed the hypermetabolic FDG uptake within the patella, which was almost the same as previous findings (6, 7). Additionally, no other sites of involvement were found in the whole-body PET/CT scan; thus, the primary malignant tumor of the patella was considered.

Histologically, PLBs include two subtypes (B-cell NHLs and T-cell NHLs); among them, DLBCLs are predominant (54–92%) in primary bone lymphomas (12). Here, our case was diagnosed with DLBCL. Besides, DLBCLs are divided into prognostically important subgroups with germinal center B-cell-like (GCB), activated B-cell-like (ABC), and type 3 gene expression profiles (13). According to the results of immunohistochemistry staining, GCB and non-GCB subtypes of DLBCL can be determined. Our case was diagnosed with non-germinal center DLBCL because of positive MUM-1 and negative CD10 and Bcl-6. It is reported that the majority of cases with DLBCL exhibit a good prognosis (14). However, patients with non-GCB may have worse overall survival than GCB (7). Thus, long-term surveillance for our case should also be considered to monitor overall survival.

Until recently, the treatment for DLBCL has been challenging and debatable. Usually, treatments for malignant PLB involve chemotherapy and localized radiation (15). However, in some similar cases, the prognosis is not satisfying despite treatment with a series of chemotherapy or radiation (3, 7). Therefore, an orthopedic surgical intervention of this disease is usually recommended before chemoradiotherapy. In order to maintain the integrity of the knee extension apparatus, curettage and bone grafting are preferred for most Enneking stage 1/2 benign tumors or tumor-like lesions of the patella. Conversely, patellectomy is suitable for the Enneking stage 3 and malignant tumors (16). Notably, Yokoyama et al. previously proposed that patellectomy before chemotherapy and radiation could also be performed in patients with isolated lesions (17). In addition, a recent prospective study has confirmed that complete surgical resection is beneficial for patients with early-stage DLBCL because complete resection minimizes tumor mass and induces a positive impact on outcome (18). As previously reported, a patient with DLBCL in the hip achieved a favorable outcome by multimodal therapy of R-CHOP chemotherapy, radiotherapy, and surgical management (19). Thus, complete resection could be worth considering when the affected area can be removed without jeopardy. Accordingly, since our patient had an isolated lesion, she underwent a patellectomy before chemotherapy to improve the prognosis. Nevertheless, due to the specific localization of the patella, the patellectomy would destroy the knee-extension apparatus and reduce the strength of the quadriceps femoris muscle, which in turn affects the functions of the knee joint. Therefore, except for the survival outcome, functional recovery is a high priority for these patients who underwent patellectomy. Theoretically, the reconstruction of knee-extension apparatus is required when necessary but controversial for the functional recovery (20). Therefore, despite no reconstruction in our case, the expansion of quadriceps femoris muscle and the residual support structure surrounding the patella was directly sutured after the patella was completely resected to improve the functional recovery of the knee.

Currently, R-CHOP is recommended as the first-line treatment for DLBCL (15). Thus, the patient was administrated with eight cycles of R-CHOP chemotherapy and achieved remission. Considering the knee function would be affected by patellectomy, the affected limb was fixed with an extension brace for 6 weeks; meanwhile, the patient received functional training and weight-loaded walking training. At 1-year follow-up, our case obtained a satisfactory functional recovery with the knee joint flexion/extension muscle strength of 5 levels, AKS of 95 points, and ROM of 0–135°. However, up to now, there is only one report about the clinical assessment of patellar functionality in the case of patella PBL. The patient’s chronic pain and ROM were resolved successfully after iliac crest grafting and chemotherapy (5). Based on these two cases, we assumed that surgical management before chemotherapy has a limited impact on functional recovery. Indeed, due to the small number of cases, the effect of multimodal therapy of R-CHOP chemotherapy plus surgical management on patellar functionality should be discussed further.

## Summary

In this case, we systematically reported the diagnosis, treatment, and prognosis of one patient with DLBCL in the patella. The patient achieved a favorable prognosis and satisfactory functional recovery from the patellectomy plus R-CHOP chemotherapy, which provides additional evidence for the standard of care.

## Data Availability Statement

The original contributions presented in the study are included in the article/supplementary material. Further inquiries can be directed to the corresponding author.

## Ethics Statement

The studies involving human participants were reviewed and approved by Shengli Oilfield Central Hospital. The patients/participants provided their written informed consent to participate in this study. Written informed consent was obtained from the individual(s) for the publication of any potentially identifiable images or data included in this article.

## Author Contributions

All authors made a significant contribution to the work reported, whether that is in the conception, study design, execution, acquisition of data, analysis and interpretation, or in all these areas; took part in drafting, revising, or critically reviewing the article; gave final approval of the version to be published; have agreed on the journal to which the article has been submitted; and agreed to be accountable for all aspects of the work.

## Conflict of Interest

The authors declare that the research was conducted in the absence of any commercial or financial relationships that could be construed as a potential conflict of interest.

## Publisher’s Note

All claims expressed in this article are solely those of the authors and do not necessarily represent those of their affiliated organizations, or those of the publisher, the editors and the reviewers. Any product that may be evaluated in this article, or claim that may be made by its manufacturer, is not guaranteed or endorsed by the publisher.
